# Effects of low-volume functional and running high-intensity interval training on physical fitness in young adults with overweight/obesity

**DOI:** 10.3389/fphys.2024.1325403

**Published:** 2024-01-31

**Authors:** Meng Cao, Baiquan Yang, Yucheng Tang, Chun Wang, Lijun Yin

**Affiliations:** ^1^ Sports College, Shenzhen University, Shenzhen, China; ^2^ School of Humanities and Social Sciences, The Chinese University of Hong Kong, Shenzhen, China

**Keywords:** obesity, physical fitness, high-intensity interval training, functional training, young adults

## Abstract

**Objectives:** This study examined and compared the effects of functional and running high-intensity interval training (HIIT) on body composition, cardiorespiratory fitness, and muscular fitness of young adults with overweight or obesity.

**Methods:** Forty-five participants (22.1 ± 2.1 years, BMI = 25.2 ± 1.0 kg/m^2^) were assigned to functional HIIT (HIIT-F; *n* = 15), running HIIT (HIIT-R; *n* = 15), or non-training control group (CON; *n* = 15). Participants in HIIT-F and HIIT-R performed functional exercise based-HIIT (four sets of all-out whole-body exercises including jumping jacks, squats, twist jumps and mountain climbers, et al.) and running HIIT (four sets of running on a treadmill) for 12 weeks, respectively. Body composition, muscular fitness, and cardiorespiratory fitness were assessed pre and post intervention.

**Results:** Both HIIT-F and HIIT-R significantly improved the body composition and cardiorespiratory fitness, with HIIT-F induced greater improvements in lean mass (+1.623 vs. −1.034 kg, *p* < 0.001), back strength (+6.007 vs. +3.333 kg, *p* < 0.01), and push-ups (+5.692 vs. 1.923 reps, *p* < 0.001) than that in HIIT-R. HIIT-R reduced more visceral fat area (VFA) (−11.416 vs. −4.338 cm^2^, *p* = 0.052) and induced similar improvement in cardiorespiratory fitness (VO_2max_, +2.192 vs. +2.885 mL/kg/min, *p* = 0.792) with HIIT-F.

**Conclusion:** Twelve weeks of HIIT-R or HIIT-F improved physical fitness among young adults with overweight or obesity. Despite the similar impact on cardiorespiratory fitness, HIIT-F generates a better positive effect on muscular fitness relative to HIIT-R, which could be partly explained by the greater increase in lean mass after HIIT-F intervention.

## 1 Introduction

A sedentary lifestyle is widely accepted as a major risk factor for the development of adiposity. The increasing prevalence of overweight and obesity is a significant public health concern worldwide. Individuals with obesity may experience a decline in muscle mass and function, leading to mental health issues and an increased risk of all-cause mortality (Sizoo et al., 2021; Kauffman et al., 2022). For instance, sarcopenic obesity, a complex condition constituting the coexistence of skeletal muscle mass loss and excess adiposity, is considered a severe medical condition associated with diminished quality of life and increased risk of mortality (Axelrod et al., 2023). Limited environments, costs, facilities, and lack of time and variation are the most common barriers to engaging in regular exercise (Bartlett et al., 2011; Kupila et al., 2023). Therefore, alternative, adaptable, and affordable exercise options that do not require traditional gym facilities are necessary.

High-intensity interval training (HIIT) involves repeated bouts of high-intensity exercise (ranging from 85 to 250% VO_2max_ for 6 s to 4 min) interspersed by low-intensity recovery (ranging from 20 to 40% VO_2max_ for 10 s to 5 min) or rest. Increasing evidence demonstrated that HIIT is a low-cost and time-efficient exercise method for improving body composition and cardiometabolic health of obese populations (Vella et al., 2017; Cao et al., 2022). However, traditional HIIT modalities (i.e., running, cycling, or rowing) still lack convenience which could cause a negative impact on exercise adherence (Desai et al., 2008). An emerging functional exercise-based HIIT (HIIT-F) program has attracted intense attention over the past few years. Unlike traditional HIIT, functional HIIT maintains the HIIT characteristics in terms of training parameters but prioritizes more functional and strength-oriented training protocols, instead of purely endurance training. These functional exercises typically include the whole body, and universal motor recruitment patterns in multiple planes of movement such as squats, deadlifts, cleans, snatches, pull-ups, and vertical jumps (Feito et al., 2018a). Several studies have shown that HIIT-F represents a more suitable and efficient exercise modality with its benefits on cardiorespiratory fitness and musculoskeletal health in young adults (Scoubeau et al., 2022; Lu et al., 2023). However, only a limited number of studies have investigated the effects of HIIT-F on young adults with overweight or obesity. For instance, previous evidence has revealed that 10 months of functional HIIT decreased the body fat and increased the fat-free mass and muscle strength in overweight/obese females and the home-based HIT improved the cardiorespiratory fitness and insulin sensitivity in obese adults (Batrakoulis et al., 2012; Scott et al., 2019; Eskandari et al., 2020). A recent study has compared the effectiveness of HIIT combined with resistance training or aerobic exercise on the immunomodulatory adaptations in young females with overweight and obesity (Soltani et al., 2023). But none of these studies concurrently compared the different impacts of functional HIIT and traditional HIIT on the physical fitness in school settings. Physical fitness comprises body composition, cardiorespiratory fitness, and muscular fitness, which are reliable indicators to measure one’s ability to perform physical activity and exercise, and determine health status (Tomkinson et al., 2018).

Therefore, the purpose of this study was to compare low-volume functional exercise-based HIIT (HIIT-F) and running-based HIIT (HIIT-R) on body composition, cardiorespiratory fitness, and muscular fitness in young adults with overweight or obesity. Based on the literature discussed, we hypothesized that body composition and VO_2max_ would be improved in both groups, but muscular fitness would be significantly improved only after HIIT-F.

## 2 Materials and methods

### 2.1 Participants and study design

**FIGURE 1:**
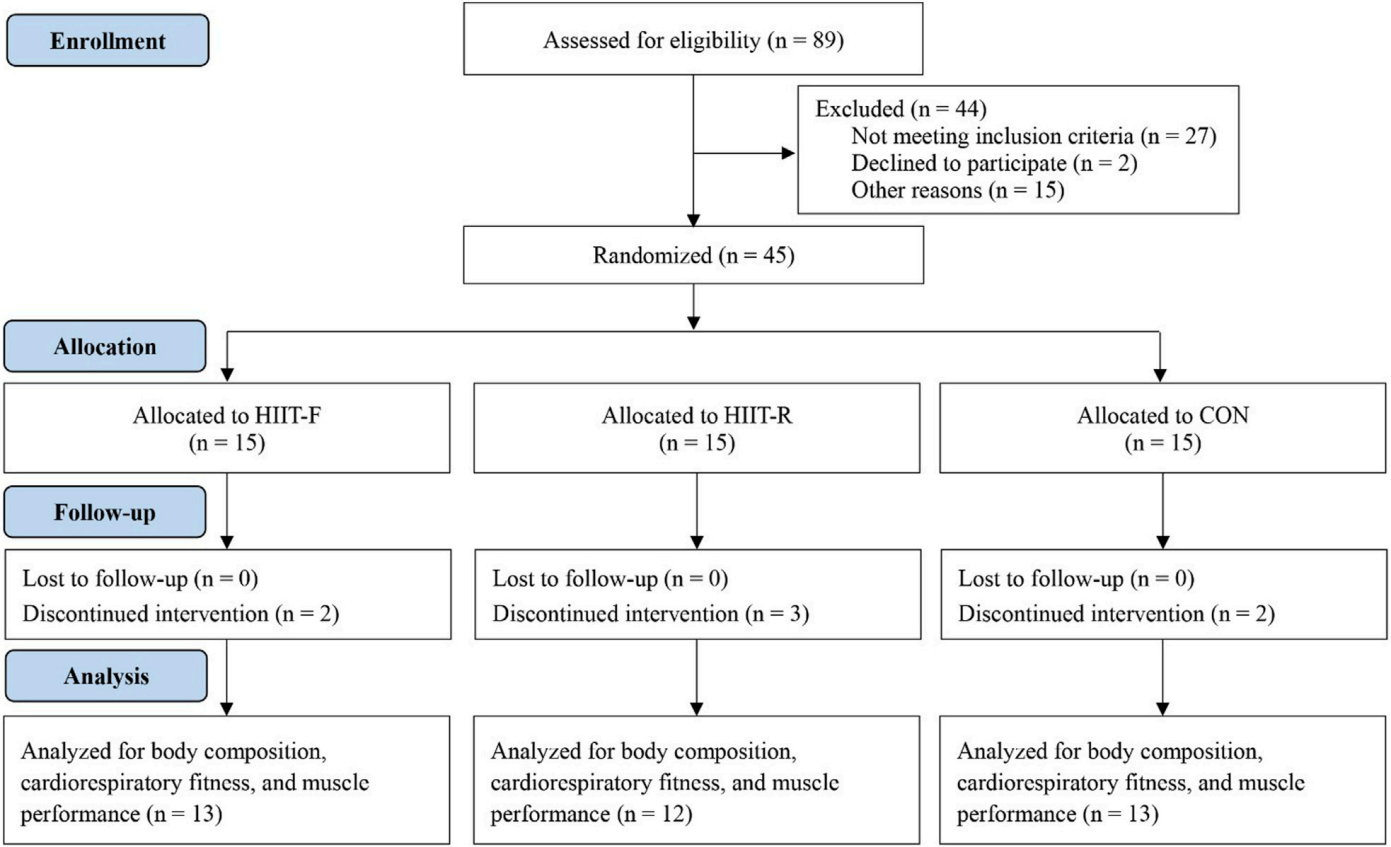
Participants Flowchart. Abbreviations: HIIT-F, functional high-intensity interval training; HIIT-R, running high-intensity interval training; CON, no-training control.

Forty-five overweight or obese adults aged 18–30 years (23 males, 22 females) participated in the study. Participants were recruited from a university via WeChat, the school website, and University email advertisements. The inclusion criteria were: 1) aged 18–30 years; 2) provided written informed consent; 3) BMI was greater than or equal to 24.0 kg/m^2^ (BMI cutoffs of 24.0 kg/m^2^ to define overweight and 28.0 kg/m^2^ to define obesity) (Zhou, 2002); and 4) had no physical limitations to exercise (e.g., cardiac abnormalities, hypertension, diabetes, orthopedic, or neuromuscular disorders). Exclusion criteria were: 1) being involved in structured exercise training within the last 6 months; 2) having a condition limiting participation to maximal physical tests and training (e.g., cardiovascular or lung disease, neuromuscular or musculoskeletal disorder). This trial was registered on the Chinese clinical trial registry (ChiCTR2100048737), conducted according to the guidelines of the Declaration of Helsinki, and approved by the medical ethics committee of the Department of Medicine of Shenzhen University (PN-2020-045). This study was conducted between September 2022 and June 2023. A flowchart and study design of this study is depicted in Figure 1, and the characteristics of the participants at baseline are detailed in Table 1.

**TABLE 1:** Characteristics of participants at baseline (Mean ± SD).

	HIIT-F (*n* = 13)	HIIT-R (*n* = 12)	CON(*n* = 13)
Age (year)	23.15 ± 2.27	21.54 ± 1.79	21.52 ± 1.82
Sex (male/female)	8/5	7/5	9/4
Weight (kg)	72.17 ± 7.15	71.13 ± 6.10	71.78 ± 4.43
BMI (kg/m^2^)	25.60 ± 1.56	25.27 ± 0.56	24.78 ± 0.56
Lean mass (kg)	28.08 ± 4.53	28.53 ± 5.43	28.37 ± 4.93
Body fat (%)	34.63 ± 4.98	38.40 ± 4.76	35.23 ± 4.98
HR max (b.p.m)	195.92 ± 3.28	195.31 ± 4.63	194.00 ± 3.34
VO_2_max (mL/kg/min)	22.44 ± 4.73	25.12 ± 4.32	25.27 ± 4.05

Notes: HIIT-F, functional high-intensity interval training; HIIT-R, running high-intensity interval training; CON, no-training control; BMI: body mass index; HR_max_, maximal heart rate; bpm: beats per minute, VO_2max_, maximal oxygen uptake.

### 2.2 Randomized

Participants were randomized 1:1:1 to HIIT-F (*n* = 15), HIIT-R (*n* = 15), or CON (*n* = 15), using a random number generator (v20.0; SPSS Inc., Chicago, IL, United States). The principal investigator (C.M.) performed the randomization of each participant after completing laboratory pre-assessments.

### 2.3 Anthropometry and body composition

Participants were instructed to fast for 10 h before anthropometric and body composition measurement. The standing height (in cm to the nearest 0.5 cm) was measured without shoes using a wall-mounted scale. Body mass (BM), body mass index (BMI), body fat percentage (%BF), Fat mass (FM), Muscle mass (MM), and estimated visceral fat area (VFA) were analyzed by bioelectrical impedance analysis (BIA). BIA can be a reliable tool for measuring body composition and VAT, the reliability has been widely verified. As previous studies suggested (Kamari et al., 2023; Vereczkei et al., 2023), we utilized Inbody 770 Body Composition Monitor (Biospace Co., Seoul, Korea) to obtain foot-to-foot BIA measures under the manufacturer’s guidelines, with participants standing barefoot on the footplates. Before the measurement, all the participants entered their sex, age, and height (cm). Furthermore, to ensure the accuracy of measurement, each subject was measured three times and the average was calculated.

### 2.4 Muscular endurance

Participants’ muscular endurance was measured using push-ups (female participants perform knee push-ups). After the warm-up exercise, participants start the test in a proper push-up position with the body lifted from the floor. Upon descent, the upper body had to touch the floor and hands had to be lifted for a second to ensure the body was completely flat on the floor. One repetition was counted when the body moved back to the starting position. The number of completed push-ups was recorded.

### 2.5 Muscular power

The power and explosive strength were measured using the standing long jump. The participants wore sneakers and stood behind the starting line with their feet placed naturally at shoulder width apart. Began testing, participants were instructed to bend their knees, swing their arms, and jump with both feet at the same time (Dransmann et al., 2021). The jumping distance measured in centimeters was recorded, and the best of three jumps was used to determine lower limb performance.

### 2.6 Grip strength

Grip strength was measured by an adjustable spring-loaded digital hand dynamometer (EH101, CAMRY, Guangdong, China) with a resolution of 0.1 kg. In each measurement, the Knob was adjusted to the appropriate position according to the size of the participant’s hand and squeezed the handle as hard as possible for approximately 3 s, three attempts were completed for a dominant hand with 30 s resting intervals between measurements. The highest measurement was recorded (Lockie et al., 2020).

### 2.7 Maximal back muscle strength

The back strength test was conducted using the electronic back strength meter (BCS-400, HFD Tech Co., Beijing, China). Participants were instructed to stand upright on the back strength meter’s chassis with both arms and hands straight and hanging down in front of the same side of the thigh. This position ensured that the handle was in contact with the tips of the two fingers, and the chain length was fixed at this height. During the test, participants were asked to straighten both legs and tilt their upper body slightly forward, about 30°. Then they were instructed to straighten both arms, tightly grip the handle with their palms facing inward, and exert maximum force by pulling upward. The highest recorded value in kilograms was considered the maximum strength achieved.

### 2.8 Cardiorespiratory fitness

Cardiorespiratory fitness (CRF) and maximal aerobic speed (MAS) were measured by a multistage 20-m shuttle run test (20-m SRT). The 20-m SRT is widely used to test the cardiorespiratory fitness and has been validated as a reliable predictor of maximum oxygen uptake (VO_2max_) (Leger and Lambert, 1982; Huhtiniemi et al., 2023; Luo et al., 2023). Compared to traditional methods of assessing VO_2max,_ the 20-m SRT is simple, easy to administer and not too time-consuming, it requires minimal equipment, and a large number of individuals can be tested simultaneously (Mayorga-Vega et al., 2015). Participants performed the test on an outdoor track between two lines that were separated by 20 m while keeping pace with the audio signals that were emitted from an MP3 that was produced by the National Coaching Foundation. The initial speed was set at 8.5 km/h and increased by 0.5 km/h every minute. The test was ended if the participants gave up or failed to run a 20 m run within the allotted time on two consecutive attempts. The last stage’s speed was considered the MAS (km/h). The VO_2max_ was calculated using the equation by previous study (Mahar et al., 2011). VO_2max_ (mL/kg/min) = 41.76799 + (0.49261 × laps) − (0.00290 × laps^2^) − (0.61613 × BMI) + (0.34787 × sex × age), 1 if male or 0 if female, and age is in years. The maximal heart rate (HR_max_) was also measured during the pre- and post-training periods.

### 2.9 The HIIT intervention protocol

HIIT-F and HIIT-R performed three sessions on non-consecutive days per week for 12 weeks. A 3 min of warm-up or cool-down movement was performed immediately before or post training. The HIIT-F content was integrated by investigators based on a previous study (Scoubeau et al., 2023) and provided to participants through four videos created by our team. Participants completed four sets of exercises in a single session, with each set consisting of 4 × 30 s all-out whole-body exercises followed by a 30-s rest. There is a 1 minute of rest between each set. Each exercise was proposed with a basic (one to two sets) and advanced variant (three to four sets) to promote progression (Table 2). Participants in the HIIT-R group performed 4 sets in one session on a treadmill, with each set including 4 × 30 s exercises followed by a 30-s rest. There is a 1 minute of rest between each set. The intensity of running was at 100% MAS for 1–4 weeks, and progress at 110% MAS for 5–8 weeks and 120% MAS for 9–12 weeks. The intensity during the recovery period was at 50% MAS. The training intensity during the whole exercise intervention period was monitored by continuous heart rate (HR, Polar team Oh1, Polar, Kemele, Finland). Besides, the average and maximal HR of each session were recorded. The total duration of each session of both HIIT-F and HIIT-R was about 25 min. The participants in the HIIT-F group completed their exercise routine on the sport court, while those in the HIIT-R group performed their intervention in the gym. Throughout their respective workouts, all participants were closely monitored by experienced practitioners.

**TABLE 2:** Protocol of the functional HIIT intervention.

Week	Sessions/week	Number of sets (4 × 30-s)	Exercises
1–4	4	1–2	1. Jumping jacks
			2. Squats
			3. Twist jumps
			4. Mountain climbers
		3–4	1. Low jacks
			2. Squat jumps
			3. Twist jumps at a faster pace
			4. Mountain climbers at a faster pace
5–8	4	1–2	1. Low jacks
			2. Plank jacks
			3. Lunges with a knee lift
			4. Knee raises with trunk rotation
		3–4	1. Squat jumps
			2. Plank jacks at a faster pace
			3. Back lunge jumps
			4. Mountain climbers at a faster pace
9–12	4	1–2	1. Jumping jacks
			2. Low-impact burpees
			3. Squats
			4. High knees
		3–4	1. Low jacks
			2. Burpees without vertical jump
			3. Squat jumps
			4. High knees at a faster pace

### 2.10 Dietary and exercise control

All the participants performed the validated 24-h dietary recalls (3 weekdays and 1 weekend day) at the initial and the end of the training program to estimate the daily energy intake (Wong et al., 2012). Commercial software (Boohee Health software, Boohee Info Technology Co., Shanghai, China) was applied to calculate the average energy intake, which was presented as kilocalories per day (kcal/day). Subjects were asked to maintain their current diet and physical activity level throughout the study. They are asked to abstain from any other form of physical training.

### 2.11 Statistical analysis

The sample size was estimated based on a previous study on interval training. Additionally, based on an estimated β = 0.8, moderate effect size f = 0.25, and a correlation among repeated measures of 0.8 for VO_2max_, a total sample size of 21 participants was calculated using G*Power 3.1 (Cao et al., 2022). All analyses were performed using the SPSS Statistical Software Package (v20.0; SPSS Inc., Chicago, IL, United States). Distributional assumptions were verified using the Kolmogorov–Smirnov test, and non-parametric methods were utilized where appropriate. All data passed the normality and homogeneity tests. An ANOVA repeated measures test was used to compare the baseline data of the three groups and to compare changes in the different variables between groups. A two-way analysis of variance (ANOVA) with repeated measures (3 groups: HIIT-F vs. HIIT-R vs. CON × 2 times: pre-vs. post-intervention). A *post hoc* test (with Bonferroni) was applied if the main factor was significant.

## 3 Results

### 3.1 Effects of HIIT-F and HIIT-R on body composition of participants

We recorded the attendance of participants in HIIT-F and HIIT-R groups. The average attendance percent of HIIT-F is 96.4%, and the average attendance percent of HIIT-R is 93.3%. The energy intake was not significantly altered in any group during the study nor different between groups, according to the validated 24-h dietary recalls. The body composition and energy intake data are presented in Table 3. The interaction effects between group and time in all the indicators were found. Compared with their relative baselines, the body weight (*p* < 0.001), body fat percent (*p* < 0.001), and VFA (*p* < 0.05) in HIIT-F and the body weight (*p* < 0.001), body fat percent (*p* < 0.001), and VFA (*p* < 0.001) in HIIT-R group were significantly decreased, and the lean mass of HIIT-F was increased (*p* < 0.001) after 12 weeks of training. No significant change in all these indicators was found in the CON group. Compared with the CON group, noticeably lower body weight (*p* < 0.05) was observed in the HIIT-R group during the post-training period. In addition, significantly lower body fat percent was observed in the HIIT-F group than in the HIIT-R group (*p* < 0.05).

**TABLE 3:** Body composition and energy intake of participants at pre- and post-training period (Mean ± SD).

	HIIT-F (*n* = 13)		HIIT-R (*n* = 12)		CON (*n* = 13)		*p*-value		
	Pre-Training	Post-Training	Pre-Training	Post-Training	Pre-Training	Post-Training	time	group	time x group
Weight (kg)	72.17 ± 7.15	70.04 ± 6.86^***^	71.13 ± 6.10	67.93 ± 6.25^***^^#^	71.78 ± 4.43	71.98 ± 4.40	0.000	0.533	0.000
Lean mass (kg)	28.08 ± 4.53	29.70 ± 5.11^***^	28.53 ± 5.43	27.49 ± 5.39^**^	28.37 ± 4.93	28.75 ± 5.41	0.021	0.899	0.000
Body fat (%)	34.63 ± 4.98	32.35 ± 4.50^***^^and^	38.40 ± 4.76	36.38 ± 4.23^***^	35.23 ± 4.98	35.40 ± 5.41	0.000	0.095	0.000
VFA (cm^2^)	76.25 ± 19.2	71.92 ± 14.98^*^	84.93 ± 18.33	73.52 ± 15.12^***^	67.55 ± 27.29	68.16 ± 30.35	0.001	0.470	0.001
EI(kcal/d)	3528.9 ± 433	3537.7 ± 387	3356.3 ± 264	3248.3 ± 259	3475.0 ± 382	3430.1 ± 365	0.266	0.227	0.543

Note: VFA, visceral adipose tissue area; EI, energy intake. ^**^ *p* < 0.01, ^***^ *p* < 0.001 vs. their relative groups at pre-training; ^#^ *p* < 0.05 vs. CON group at post-training; ^&^ *p* < 0.05 vs. HIIT-R group at post-training.

**FIGURE 2:**
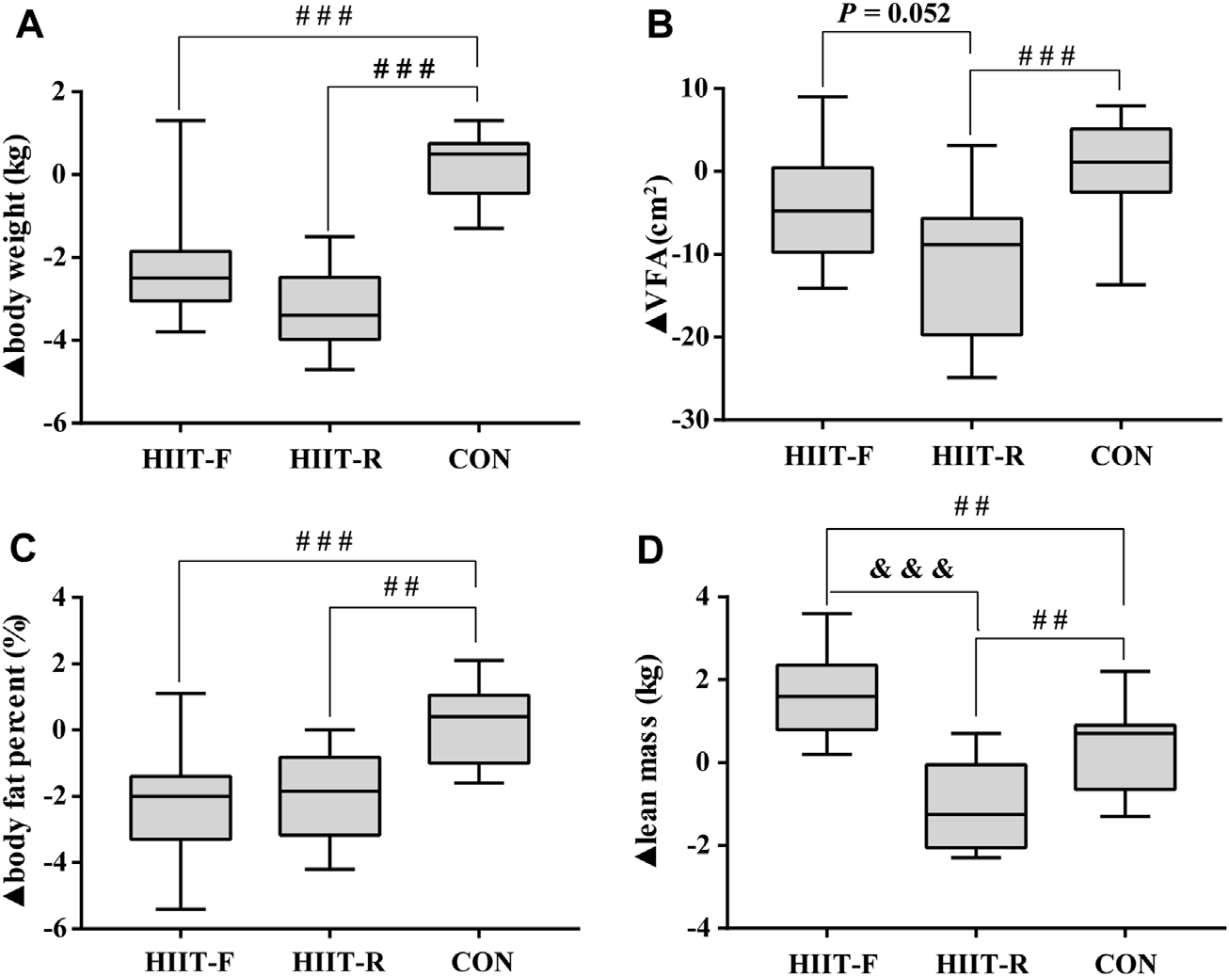
The change magnitudes of body weight **(A)**, VFA **(B)**, body fat percent **(C)**, and lean mass **(D)** after 12 weeks of intervention. Note: ▲: The difference between post-training and baseline (▲ = post-baseline). ^##^ *p* < 0.01, ^###^ *p* < 0.001 vs. CON group; ^&&&^ *p* < 0.001 vs. HIIT-R group.

We also compared the change magnitude of each indicator after a 12-week intervention. As Figure 2 shows, the decreasing magnitudes in body weight (*p* < 0.001, *p* < 0.001), body fat percent (*p* < 0.001, *p* < 0.01) in HIIT-F and HIIT-R group, and VFA (*p* < 0.001) in HIIT-R group were significantly greater than that in CON group. Additionally, both HIIT-R and HIIT-F increased more lean mass (*p* < 0.01, *p* < 0.01) than CON. When compared with the HIIT-R group, the greater change magnitude in lean mass (*p* < 0.001) was observed. Interestingly, we found the typical different adaptation to a standardized exercise in HIIT-R group, in which the VFA of participant #8 was increased by 3 cm^2^, while participant #1 (same sex with similar BMI at baseline) showed a significant decrease in VFA by 24.9 cm^2^. Other outcomes including VO_2max_, lean mass, standing long jump, grip force, and back strength of participant #1 are better than participant #8. These results suggest that either HIIT-F or HIIT-R generates significant benefits for body composition, with a more beneficial effect on the lean mass by HIIT-F and on the VFA by HIIT-R intervention.

### 3.2 Effects of HIIT-F and HIIT-R on muscular fitness of participants

The muscular fitness was reflected by muscular endurance, power, and strength. As Table 4 shows, there is an interaction effect between group and time in all indicators. Compared with their relative baselines, the standing long jump (*p* < 0.001), push-ups (*p* < 0.001), hand grip (*p* < 0.001), and back strength (*p* < 0.001) in the HIIT-F and the standing long jump (*p* < 0.001), push-ups (*p* < 0.001), hand grip (*p* < 0.001), and back strength (*p* < 0.001) in the HIIT-R groups were dramatically increased after 12 weeks of training. CON group during the post-training period showed significant increases in push-ups (*p* < 0.01), hand grip (*p* < 0.01), and back strength (*p* < 0.001), compared with that at baseline.

**TABLE 4:** Muscular fitness of participants at pre- and post-training period (Mean ± SD).

	HIIT-F (*n* = 13)		HIIT-R (*n* = 12)		CON (*n* = 13)		*p*-value		
	Pre-Training	Post- Training	Pre-Training	Post- Training	Pre-Training	Post- Training	time	group	time x group
SLJ (cm)	192.31 ± 20.71	198.31 ± 21.33^***^	191.08 ± 23.81	195.42 ± 22.61^***^	190.38 ± 24.73	190.69 ± 24.94	0.000	0.849	0.000
Push-up (reps)	25.38 ± 8.29	31.08 ± 9.00^***^	23.92 ± 7.47	25.85 ± 8.14^***^	24.54 ± 8.01	25.69 ± 7.83^**^	0.000	0.513	0.000
Hand grip (kg)	37.57 ± 7.71	40.35 ± 7.83^***^	36.02 ± 9.83	38.03 ± 9.78^***^	38.66 ± 8.57	40.02 ± 8.24^**^	0.000	0.761	0.042
Back force (kg)	37.31 ± 10.84	43.38 ± 11.42^***^	35.83 ± 12.36	39.17 ± 12.53^***^	37.54 ± 11.10	39.85 ± 11.15^***^	0.001	0.815	0.001

Note: SLJ, standing long jump. ^**^ *p* < 0.01, ^***^ *p* < 0.001 vs. their relative groups at pre-training.

**FIGURE 3:**
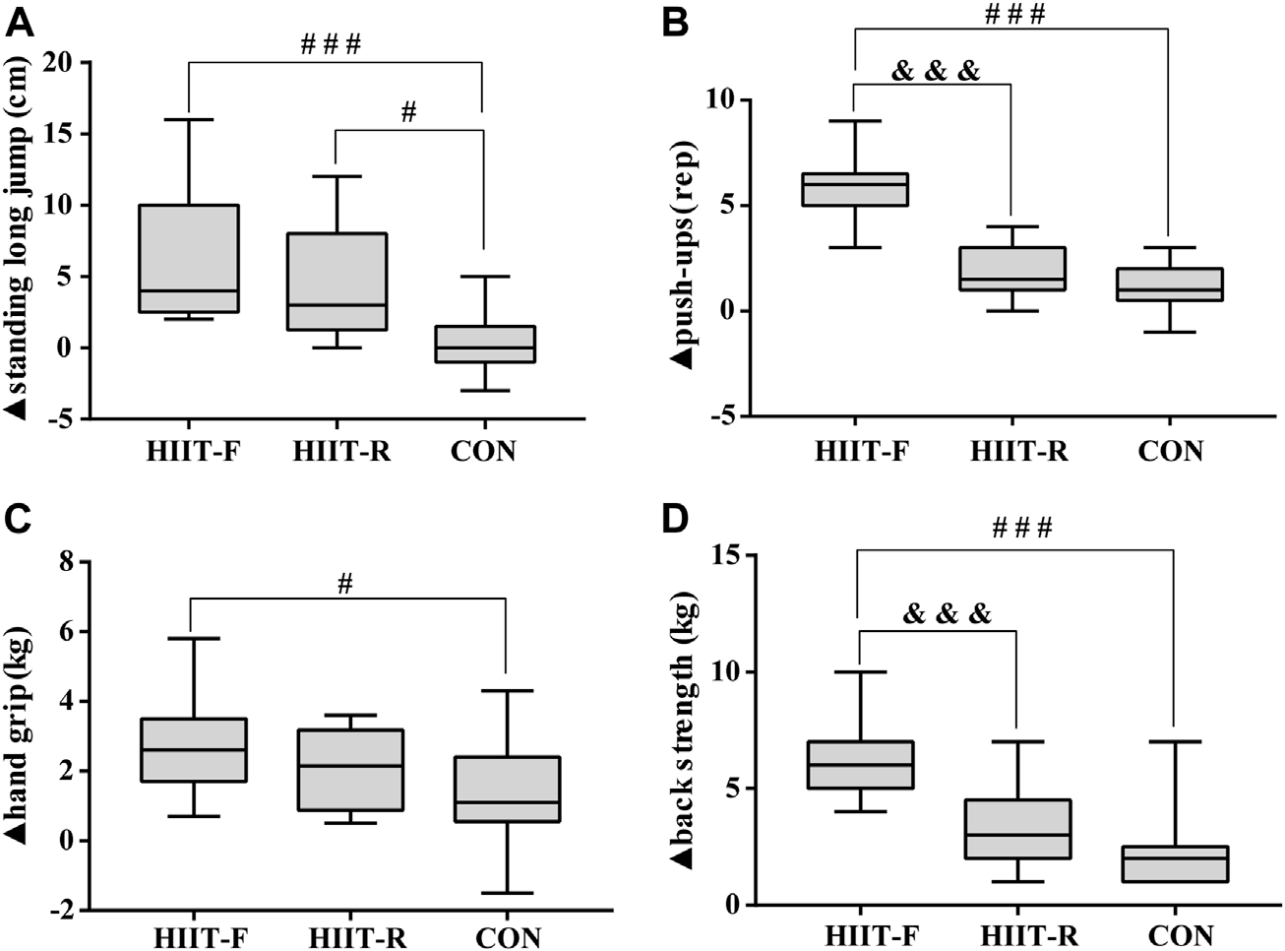
The change magnitudes of standing long jump **(A)**, push-ups **(B)**, hand grip **(C)**, and back strength **(D)** after 12 weeks of intervention. Note: ▲: The difference between post-training and baseline (▲ = post-baseline). ^#^ *p* < 0.05, ^###^ *p* < 0.001 vs. CON group; ^&&&^ *p* < 0.001 vs. HIIT-R group.

Figure 3 shows the change magnitude of muscular fitness during the post-training period. We observed the significantly greater change magnitude of standing long jump (*p* < 0.001), push-up (*p* < 0.001), hand grip (*p* < 0.05) and back strength (*p* < 0.001) in HIIT-F group, and standing long jump (*p* < 0.05) in HIIT-R group, compared with CON group. Additionally, a more superior effect on push-up (*p* < 0.001) and back strength (*p* < 0.001) were found in HIIT-F, compared with the HIIT-R group. These results indicate that although muscular fitness has improved after a 12-week intervention in all groups, the change magnitudes vary among groups, with the HIIT-F exercise generating the most superior effect on boosting muscular fitness.

### 3.3 Effects of HIIT-F and HIIT-R on cardiorespiratory fitness of participants

We determined the 20 m-SRT, MAS, HR_max_, and VO_2max_ to indicate the cardiorespiratory fitness of participants. As Table 5 shows, an interaction effect between group and time was found for 20 m-SRT and VO_2max_. Compared with their relative baselines, 20 m-SRT (*p* < 0.001, *p* < 0.001, *p* < 0.05) and VO_2max_ (*p* < 0.001, *p* < 0.001, *p* < 0.05) were significantly increased in the HIIT-F, HIIT-R (both *p* < 0.001), and CON (both *p* < 0.05) groups at post-training period. Greater 20 m-SRT (*p* < 0.05) was obtained in the HIIT-F group, compared with the CON group at post-training. For HR_max_, no interaction effect between group and time was found, and the main effect of the group was also not statistically significant. While the main effect of time was significant (post-training was greater than pre-training, *p* < 0.024). For MAS, after a 12-week intervention, significant increases in MAS were found in the HIIT-F, HIIT-R, and CON groups, compared with their relative baseline. Greater MAS (*p* < 0.05) was also observed in the HIIT-F group than that in CON.

**TABLE 5:** Cardiorespiratory fitness of participants at pre- and post-training period (Mean ± SD).

	HIIT-F (*n* = 13)		HIIT-R (*n* = 12)		CON (*n* = 13)		*p*-value		
	Pre-Training	Post-Training	Pre-Training	Post-Training	Pre-Training	Post-Training	time	group	timex group
20m-SRT (lap)	32.54 ± 4.45	39.85 ± 4.88^***^^#^	32.83 ± 4.32	38.00 ± 3.56^***^	33.62 ± 4.78	34.92 ± 2.53^*^	0.000	0.432	0.000
MAS (km/h)	25.38 ± 8.29	31.08 ± 9.00^**#^	23.92 ± 7.47	25.85 ± 8.14^**^	24.54 ± 8.01	25.69 ± 7.83^*^	N/A	N/A	N/A
HR_max_ (b.p.m)	195.92 ± 3.28	196.15 ± 2.64	195.31 ± 4.63	196.23 ± 2.80	194.00 ± 3.34	194.54 ± 2.57	0.024	0.343	0.556
VO_2max_ (mL/kg/min)	22.44 ± 4.73	25.33 ± 4.32^***^	25.12 ± 4.32	27.32 ± 3.76^***^	25.27 ± 4.05	26.56 ± 3.12^*^	0.000	0.361	0.025

Note: 20 m-SRT, 20 m shuttle run test; MAS, maximal aerobic speed; HR_max_, maximal heart rate; VO_2max_, maximal oxygen uptake; N/A, not applicable. ^*^ *p* < 0.05, ^**^ *p* < 0.01, ^***^ *p* < 0.001 vs. their relative groups at pre-training;^#^ *p* < 0.05 vs. CON group at post-training.

**FIGURE 4:**
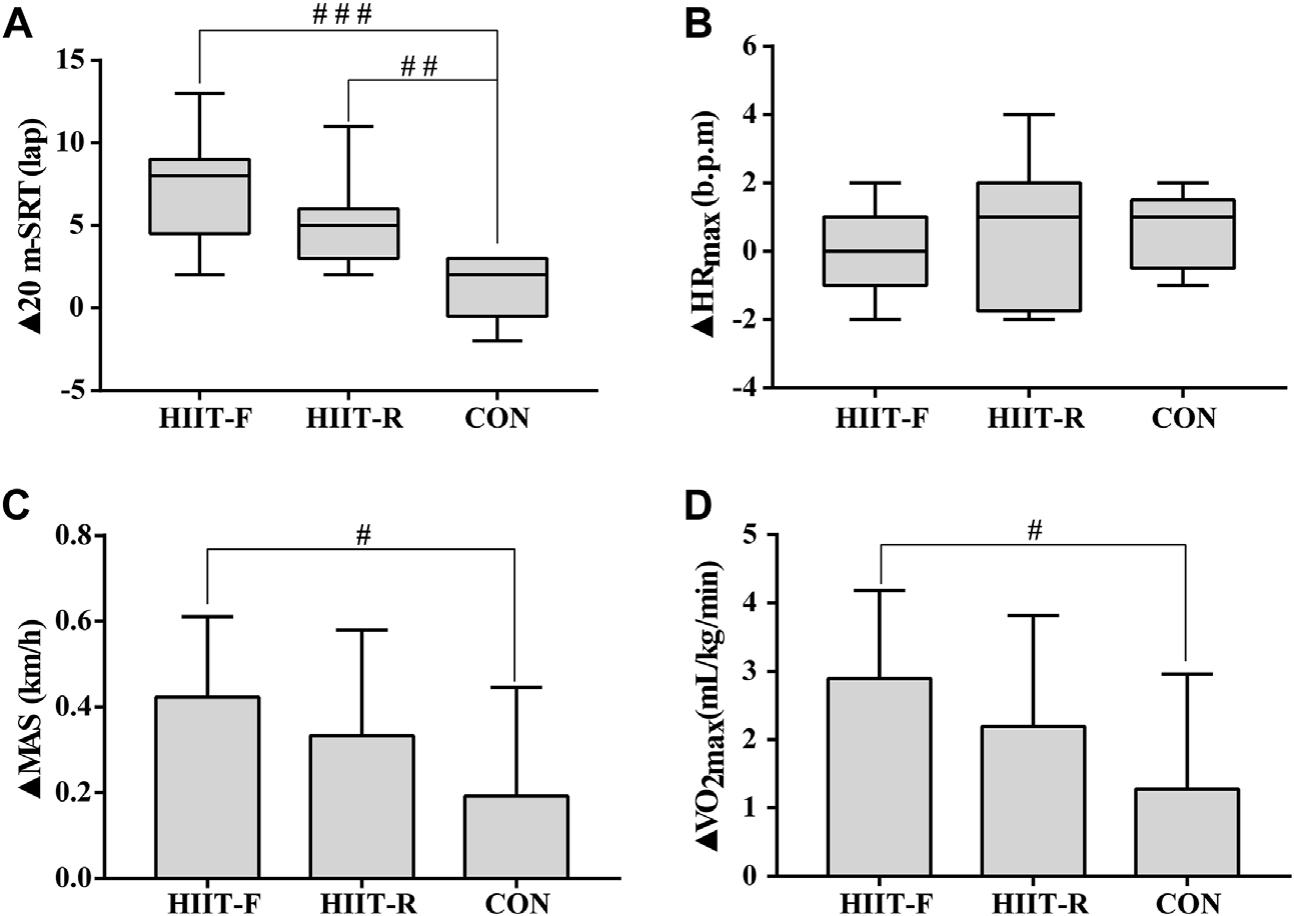
The change magnitudes of 20 m-SRT **(A)**, HRmax **(B)**, MAS **(C)**, and VO2max **(D)** after 12 weeks of intervention. Note: ▲: The difference between post-training and baseline (▲ = post-baseline). ^#^ *p* < 0.05, ^##^ *p* < 0.01, ^###^ *p* < 0.001 vs. CON group.

Figure 4 shows the change magnitude of cardiorespiratory fitness during the post-training period. We observed the significantly greater change magnitude of 20 m-SRT (*p* < 0.001), MAS (*p* < 0.05), and VO_2max_ (*p* < 0.05) in the HIIT-F group, and 20 m-SRT (*p* < 0.01) in HIIT-R group, compared with CON group. No significant difference was found in the change magnitude of HR_max_ (*p >* 0.05) among these groups.

## 4 Discussion

This study compared two regimes of high-intensity interval training (functional training-based HIIT, HIIT-F vs. running-centered HIIT, HIIT-R) on body composition and physical fitness in young adults with overweight or obesity. The main findings of the present study are that both interventions generate positive effects on body composition (body fat percentage, lean mass, and VFA), CRF (VO_2max_), and muscular fitness (hand grips, back strength, push-ups, and SLJ) of participants with different potency. In particular, the HIIT-F intervention has a more robust impact on increasing lean mass and enhancing muscular fitness, whereas HIIT-R provides greater benefits in reducing VFA. Similar potency is found between these two regimes in terms of the effect on CRF. These results largely align with our previous hypothesis.

HIIT has been widely accepted as a popular exercise regime for the efficiency and positive impacts on metabolism and physical fitness it brings. Typical HIIT regimes (e.g., HIIT-R) typically involve traditional exercise modalities such as running, cycling, and rowing (Buckley et al., 2015). However, these may result in low adherence among recreationally active individuals due to factors such as limited environments, facilities, a lack of variety (Kupila et al., 2023), and necessary supervision in the real-world setting (ROY et al., 2018). Therefore, HIIT-F has gained intense attention among various populations ranging from healthy individuals to participants with chronic diseases for its functional movement pattern, resistance-based protocol, intrinsic motivation (Heinrich et al., 2014), and benefits for metabolic profiles (Nieuwoudt et al., 2017; Fealy et al., 2018). However, to our best knowledge, a study comparing the effects of HIIT-F and HIIT-R on body composition and muscular fitness in young adults with overweight or obesity is still limited so far, and the current research aimed at broadening this knowledge. Not surprisingly, we found that both HIIT-R and HIIT-F improved body composition in young adults with overweight or obesity, which is in line with previous studies showing that body fat percentage, and visceral adipose tissue in obese adults were significantly decreased after 8–12 weeks of thrice weekly HIIT-R (Vella et al., 2017)and HIIT-F (Zhang et al., 2021). Recent studies have also demonstrated a beneficial influence of HIIT-F on body composition in individuals (Sperlich et al., 2017; Lu et al., 2021; Cao et al., 2022). Interestingly, we found a notably different efficacy between HIIT-F and HIIT-R when improving the body composition, with the more superior impact on increasing lean mass for HIIT-F and on decreasing visceral adipose tissue for HIIT-R. These results align with previous evidence indicating that aerobic training (e.g., running) is the optimal exercise mode for reducing fat mass and body mass, while a program including resistance training is needed for increasing lean mass (Willis et al., 2012). Multimodal HIIT-F including resistance training may give the added benefit of muscle mass increases (Westcott, 2012; Brown et al., 2018). It is important to note that two recent studies have indicated that after 16 weeks or 8 weeks of HIIT-F, a significant decrease in body fat percentage was observed with no changes in body mass, which is inconsistent with results in the current study showing the decreases in both body mass and fat percentage. The different change magnitude between fat mass and lean mass may explain such difference. A greater increase in lean mass was observed in these studies (Feito et al., 2018b; Lu et al., 2021), while a significantly greater decrease in VFA than increase in lean mass was found in our study. Surprisingly, we found a decreased lean mass in the HIIT-R group. The shortage of strength training or energy deficit may partially explain this (Park et al., 2023). HIIT-R can improve cardiovascular fitness and expend energy but lacks resistance training, which may not provide enough stimulus for muscle growth. Additionally, HIIT-R expends a significant amount of energy, if the individual is not consuming enough calories, especially protein to compensate for the energy expenditure, their body may start breaking down muscle tissue to meet its energy needs (Kim et al., 2022). Interestingly, we found the relative greater decrease (although not statistically different) in energy intake in HIIT-R group, compared with HIIT-F, after 12 weeks of interventions, according to the results of validated 24-h recall. These results reveal that HIIT-F generates more improvement in increasing lean mass and less efficacy in decreasing VFA than HIIT-R. Moreover, it is worth noting that it is important to preserve skeletal muscle mass while reducing fat mass, especially in individuals with sarcopenic obesity. Interventions involving resistance training and appropriate protein supply are the main ways of preserving skeletal muscle mass, as well as muscle function (Kim et al., 2022). Thus, HIIT-F could be an improved option to combat sarcopenic obesity over HIIT-R, considering its stimuli for muscle growth by resistance training elements and absence of energy deficits.

Additionally, we compared the impact of HIIT-F and HIIT-R on muscular fitness. We found that similar to HIIT-R, HIIT-F significantly enhanced muscular strength (e.g., SLJ, push-ups, hand grip, and back strength). Still, HIIT-F is superior to HIIT-R in improving the repetitions of push-ups and back strength (Figure 3). The greater increase in lean body mass (+5.8%) after HIIT-F can partly explain the improvements in muscular strength. However, considering that a decrease in lean weight accompanies the improvement in muscle fitness in the HIIT-R group, this change may result from the progression of neural adaptation and muscle coordination. Our finding is consistent with other studies. Buckley et al. reported a significant increase in muscle performance after 6 weeks of HIIT-F. In contrast, no increase was found in the HIIT group using rowing as the exercise modality (Buckley et al., 2015). The multimodal HIIT-F group increased deadlift 1RM significantly more than the rowing-based HIIT group (Brown et al., 2018). Our findings indicated that whole-body functional training to overcome self-resistance can improve muscle strength. However, the effect of HIIT-F on muscle performance varies with exercise design and test methods. In this study, when the intensity and duration of the two HIIT regimes are relatively the same, HIIT-F is more effective than HIIT-R in enhancing muscle strength. Taken together, we found that HIIT-F also generates a significant positive impact on muscular fitness like HIIT-R and even exerts more efficacy on improving push-ups and back strength tests. However, further research is needed to explore the changes in muscle fiber types and sizes between individuals caused by training.

The cardiorespiratory response to HIIT has attracted intense attention among researchers. We used 20-m SRT to estimate the VO_2max_ as previous studies suggested (Huhtiniemi et al., 2023; Luo et al., 2023). We found a significant increase in VO_2max_ among subjects after two regimes of HIIT, which is unsurprising, as previous studies have shown that HIIT based on running or cycling can significantly increase VO_2max_ (Batacan et al., 2017). In this study, subjects from both the HIIT-F and HIIT-R groups experienced improvements in VO_2max_ (12.9%, and 8.7%, respectively). In line with the recent study by Lu and others, an increase of 12.7% in the VO_2max_ was found after a low-volume HIIT-F (Lu et al., 2023). In addition, compared to the CON group, the improvement of VO_2max_ in the HIIT-F was slightly greater than that in the HIIT-R, and it should be noted that in this study, the enhanced VO_2max_ observed after HIIT-F was significantly greater than values recorded in previous studies (Brisebois et al., 2018). The following reasons could explain it: firstly, the lower baseline value in the HIIT-F (22.4 mL/kg/min); secondly, this study used a longer term (12 weeks vs. 8 weeks) to implement functional exercises; thirdly, improvements in VO_2max_ were related to the testing modality, the laps of 20 m-SRT may be easier to increase. Therefore, it is necessary to study further the effectiveness of training intensity, duration, and work recovery time ratio in improving cardiorespiratory fitness induced by HIIT. Interestingly, we have not observed the difference in efficacy on cardiorespiratory fitness among HIIT-F and HIIT-R, which is in line with previous results showing that HIIT-F achieved the same improvements in VO_2max_ and HR_max_ as HIIT-R (Menz et al., 2019). It is worth noting that the underlying mechanism by which HIIT increase VO_2max_ still remains largely elusive and will be explored in our future study, although previous study has reported that increases in VO_2max_ as a result of HIIT are mediated by improvements in central O_2_ delivery rather than peripheral adaptations (Astorino et al., 2017). To sum up, these results suggest that HIIT-F and HIIT-R play similar positive roles in improving cardiorespiratory fitness. Nevertheless, the HIIT-F may have an advantage in terms of exercise compliance, as the training may be perceived as less strenuous compared to running, a perception reflected in the results from the continuous heart rate record and attendance rate. In detail, participants in the HIIT-F and HIIT-R groups conducted their respective HIIT sessions at 78% HRmax and 80% HRmax, with attendance rates of 96.4% and 93.3%, respectively.

Some limitations of the present study should be acknowledged: 1) Tests of VO_2max_ and muscular fitness were limited to field tests. Even though 20 m-SRT with high reliability and validity was selected (Mayorga-Vega, et al., 2015), laboratory tests with an indirect calorimeter (Cosmed K5) would be more accurate. 2) The sample size of the present study is relatively small, which may lead to selection bias in the study results. The study quality could be further improved if a diary or questionnaire with an accelerometer recorded each participant’s daily physical activity level.

## 5 Conclusion

Twelve weeks of high-intensity interval training based on running or functional exercises improved body composition and cardiorespiratory fitness among young adults with overweight or obesity. Functional exercise-centered HIIT has been shown to be a more effective alternative for promoting lean mass and muscular fitness compared to running-based HIIT. Additionally, it has a similar impact on cardiorespiratory fitness, making it a promising option for combating sarcopenic obesity. Furthermore, HIIT-F can be performed anywhere with exercise compliance, limiting the barriers of needing more time/money. It may be helpful for individuals to promote physical activity and the associated benefits of a prolonged healthy lifestyle.

## Data Availability

The raw data supporting the conclusions of this article will be made available by the authors, without undue reservation.
